# Metabolism of ticagrelor in patients with acute coronary syndromes

**DOI:** 10.1038/s41598-018-29619-9

**Published:** 2018-08-06

**Authors:** Piotr Adamski, Katarzyna Buszko, Joanna Sikora, Piotr Niezgoda, Malwina Barańska, Małgorzata Ostrowska, Przemysław Paciorek, Eliano P. Navarese, Diana A. Gorog, Jacek Kubica

**Affiliations:** 1 Department of Cardiology and Internal Medicine, Collegium Medicum, Nicolaus Copernicus University, Bydgoszcz, Poland; 2 Department of Theoretical Foundations of Biomedical Science and Medical Informatics, Collegium Medicum, Nicolaus Copernicus University, Bydgoszcz, Poland; 3 Department of Pharmacology and Therapy, Collegium Medicum, Nicolaus Copernicus University, Bydgoszcz, Poland; 4 Inova Heart and Vascular Institute, Inova Center for Thrombosis Research and Drug Development, Fairfax, VA USA; 5 SIRIO MEDICINE research network, Inova Heart and Vascular Institute, Inova Center for Thrombosis Research and Drug Development, Fairfax, VA USA; 6 National Heart & Lung Institute, Imperial College, London, United Kingdom

## Abstract

Ticagrelor is a state-of-the-art antiplatelet agent used for the treatment of patients with acute coronary syndromes (ACS). Unlike remaining oral P2Y12 receptor inhibitors ticagrelor does not require metabolic activation to exert its antiplatelet action. Still, ticagrelor is extensively metabolized by hepatic CYP3A enzymes, and AR-C124910XX is its only active metabolite. A post hoc analysis of patient-level (n = 117) pharmacokinetic data pooled from two prospective studies was performed to identify clinical characteristics affecting the degree of AR-C124910XX formation during the first six hours after 180 mg ticagrelor loading dose in the setting of ACS. Both linear and multiple regression analyses indicated that ACS patients presenting with ST-elevation myocardial infarction or suffering from diabetes mellitus are more likely to have decreased rate of ticagrelor metabolism during the acute phase of ACS. Administration of morphine during ACS was found to negatively influence transformation of ticagrelor into AR-C124910XX when assessed with linear regression analysis, but not with multiple regression analysis. On the other hand, smoking appears to increase the degree of ticagrelor transformation in ACS patients. Mechanisms underlying our findings and their clinical significance warrant further research.

## Introduction

Excessive platelet activation and aggregation play essential role in the pathophysiology of acute coronary syndromes (ACS), including acute myocardial infarction (AMI)^1^. Platelet inhibition with dual antiplatelet therapy, consisting of aspirin and one of P2Y12 receptor inhibitors, is the mainstay of ACS treatment. Blockade of platelet P2Y12 receptors is particularly important in ACS patients treated with percutaneous coronary interventions (PCI), as insufficient limitation of platelet activation increases the risk of thrombotic complications and death^2^.

Ticagrelor is a state-of-the-art antiplatelet agent used for the treatment of patients with ACS. According to both European and American guidelines, it is the first line of treatment in a wide spectrum of ACS patients, including patients with ST-elevation myocardial infarction (STEMI) and moderate-to-high-risk non-ST-elevation ACS (NSTE-ACS), regardless of initial treatment strategy and including those pretreated with clopidogrel^3–6^.

Ticagrelor is a potent, reversibly binding, noncompetitive, oral P2Y12 receptor antagonist^7^. It is characterized by linear pharmacokinetics, and unlike other available P2Y12 receptor inhibitors administered orally, it does not necessitate metabolic activation to exert its antiplatelet action^8^. Still, ticagrelor is extensively metabolized by hepatic CYP3A enzymes. AR-C124910XX is the major of 10 identified ticagrelor metabolites and the only active one. Importantly, AR-C124910XX exerts at least as potent P2Y12 receptor inhibition as the parent drug^9^.

In healthy volunteers and patients with stable coronary artery disease ticagrelor is rapidly absorbed from the gastrointestinal tract and transformed into AR-C124910XX, with time to maximal concentration (t_max_) 1.3–2 h and 1.5–3 h for the parent drug and its metabolite, respectively^9–11^. Numerous clinical factors have been shown not to influence bioavailability of ticagrelor or its antiplatelet effect in healthy volunteers^12^. However, in the setting of ACS where the expeditious onset of antiplatelet action is of vast importance, the intestinal uptake of ticagrelor may be significantly delayed, especially in patients administered with morphine and subjects suffering from STEMI, with ticagrelor t_max_ delayed up to 4–5 h^13–16^. Subsequently, lagged absorption postpones formation of AR-C124910XX and the onset of platelet inhibition exerted by ticagrelor and its active metabolite^17^.

Two cytochrome P450 enzymes, CYP3A4 and CYP3A5, are responsible for the formation of AR-C124910XX^18^. Strong CYP3A inducers and inhibitors greatly affect pharmacokinetics of ticagrelor and its active metabolite, thus their co-administration with ticagrelor is contraindicated^19,20^. In healthy volunteers the bioavailability of AR-C124910XX ranges from 35% to 40% of ticagrelor plasma concentration, whereas in patients with AMI it may be as low as 21% during the initial hours of treatment^8,9,21^. Although ticagrelor may increase bioavailability of some statins, currently there are no data indicating that any of the medications routinely used for the treatment of ACS may affect metabolism of ticagrelor^22^. This also applies to morphine, the elementary analgesic used to relive chest pain in patients with ACS^21^. Therefore, it remains obscure what causes this discrepancy in the bioavailability of AR-C124910XX between healthy subjects and patients with ACS.

In the current study we sought to identify demographic and clinical factors affecting metabolism of ticagrelor into its active metabolite during the first six hours after ticagrelor loading dose (LD) in patients with ACS.

## Methods

### Study design

A post hoc analysis of integrated patient-level data pooled from two prospective, phase IV, single center, investigator-initiated trials evaluating pharmacokinetics of ticagrelor in patients with ACS (NCT02612116 - randomized, open-label, active-controlled study, n = 48; NCT02602444 - observational study, n = 73) was performed to determine whether ticagrelor metabolism in patients with ACS is affected by any of the recorded clinical characteristics. Both trials were conducted in accordance with the principles contained in the Declaration of Helsinki and Good Clinical Practice guidelines. The studies were approved by The Ethics Committee of Nicolaus Copernicus University in Toruń, Collegium Medicum in Bydgoszcz, Poland (NCT02612116 - approval number KB 540/2015; NCT02602444 - approval number KB 617/2015). Written informed consent was obtained from each patient prior to any study specific procedures. Study protocols with full lists of inclusion and exclusion criteria, detailed description of methodology, and results of the two included trials were previously published in peer-reviewed journals^14,23–25^.

In line with the available literature^8,9^, the ratio of area under the plasma concentration-time curve for AR-C124910XX (*AUC*_*M*_) to AUC for ticagrelor (*AUC*_*T*_) was chosen as a marker of the degree of ticagrelor transformation into its active metabolite $(\frac{AU{C}_{M}}{AU{C}_{T}})$. A linear regression analysis of 16 available clinical features was planned to pinpoint characteristics related to lower $\frac{AU{C}_{M}}{AU{C}_{T}}$ during the first six hours after a standard 180 mg ticagrelor LD in ACS patients. The analyzed variables included: age, gender, body mass index (BMI), obesity (BMI ≥ 30 kg/m^2^), creatinine plasma concentration at admission, glomerular filtration rate (GFR) at admission, type of ACS (STEMI vs. NSTE-ACS), type of administered ticagrelor tablets (integral vs. crushed), morphine co-administration, history of coronary artery disease, history of nonhemorrhagic stroke, left ventricle ejection fraction <50% at discharge, arterial hypertension, diabetes mellitus (DM), dyslipidemia, and current smoking (Table 1). A subsequent multiple regression analysis was intended to verify which characteristics are connected with lower $\frac{AU{C}_{M}}{AU{C}_{T}}$ during the initial period of ACS treatment with ticagrelor.

**Table 1:** Baseline characteristics of the study participants.

	n = 117
Age in years	63.5 ± 9.9
Female	40 (34)
BMI in kg/m^2^	27.8 ± 4.4
Obesity (BMI ≥ 30 kg/m^2^)	30 (26)
Creatinine at admission in mg/dL	0.87 ± 0.19
GFR at admission in mL/minute	83.3 ± 16.0
STEMI	46 (39)
NSTE-ACS	71 (61)
NSTEMI	24 (21)
UA	47 (40)
Integral ticagrelor tablets	86 (74)
Morphine administration	33 (28)
History of CAD	35 (30)
Nonhemorrhagic stroke	9 (8)
LVEF at discharge <50%	59 (50)
Hypertension	75 (64)
Diabetes mellitus	25 (21)
Dyslipidemia	104 (89)
Current smoker	43 (37)

Data are shown as mean ± standard deviation or number (%). BMI: body mass index, CAD: coronary artery disease, GFR: glomerular filtration rate, LVEF: left ventricle ejection fraction, NSTE-ACS: non-ST-elevation acute coronary syndrome, NSTEMI: non-ST-elevation myocardial infarction, STEMI: ST-elevation myocardial infarction, UA: unstable angina.

### Patient population

**Figure 1:**
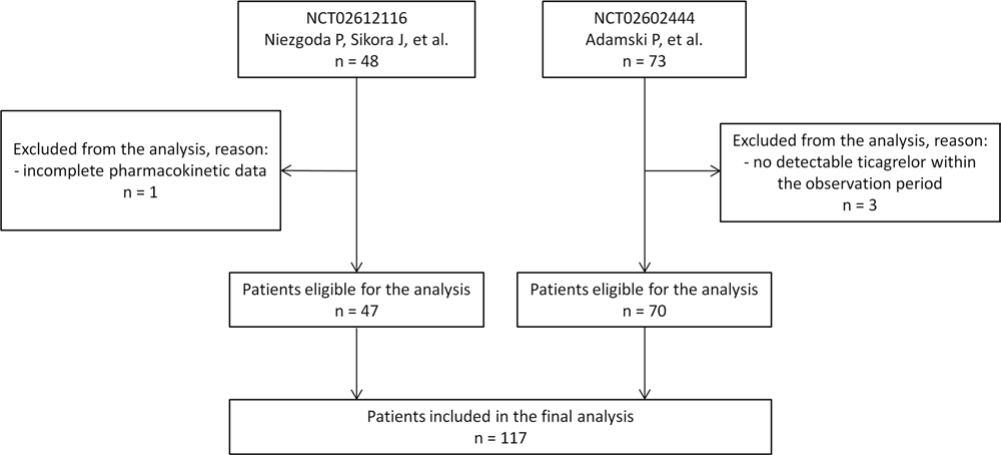
Patient flow diagram.

The diagnosis of STEMI, non-ST-elevation myocardial infarction (NSTEMI) or unstable angina (UA), and provision of informed consent for coronary angiography and PCI were required for the current analysis entry. The diagnosis of STEMI and NSTEMI was made according to the Third Universal Definition of Myocardial Infarction^26^. UA was diagnosed according to the 2015 European Society of Cardiology guidelines for the management of NSTE-ACS^4^. To avoid any potential cofactors affecting hepatic metabolism of ticagrelor, patients on therapy with any strong CYP3A inhibitors or inducers were not included in either of the studies. All subjects were P2Y12 receptor inhibitor-naive at the time of enrolment for each study, and received 180 mg LD of ticagrelor as a part of dual antiplatelet therapy with aspirin. Overall 121 ACS patients were screened, and 117 were eventually included in the analysis (Fig. 1). Patients with no detectable ticagrelor in plasma within the observation period were not included in the analysis.

### Concomitant treatment

During their participation in the trials, all patients were treated according to the European Society of Cardiology (ESC) guidelines^4,27^. Standard therapy included aspirin, beta-blockers, statins, and angiotensin-converting enzyme inhibitors or angiotensin II receptor blockers, if not contraindicated. During the periprocedural period, all study participants received unfractionated heparin in body weight adjusted dose according to the ESC recommendations. To ensure that no potential drug-drug interactions affect the results of this analysis, each patient’s individual data were screened to identify any treatment with medications known to interplay with metabolism of ticagrelor. However, after a careful inspection none of the patients from both trials was excluded from the analysis due to this reason.

### Pharmacokinetic evaluation

**Figure 2:**
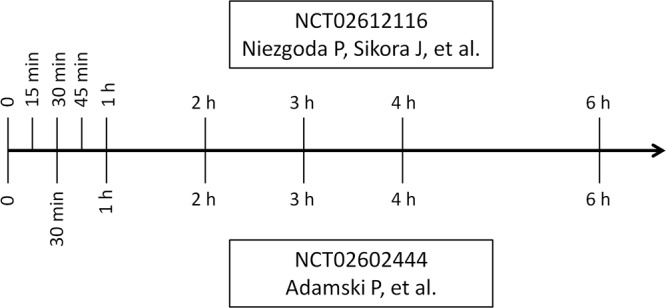
Blood sampling schedules.

Blood samples for the pharmacokinetic assessment were obtained at prespecified time points before and after 180 mg ticagrelor LD, using an 18-gauge venous catheter inserted into a forearm vein (at baseline, 30 minutes, 1, 2, 3, 4 and 6 hours after ticagrelor LD in both studies, and additionally at 15 and 45 minutes after ticagrelor LD in patients included in NCT02612116 study) (Fig. 2)^23,24^. The samples were collected into lithium-heparin vacuum test tubes and immediately after collection each sample was placed on dry ice and transferred to the central laboratory. Subsequently, within 20 min from collection, blood specimens were centrifuged at 1500 g for 12 min at 4 °C. Within 10 min postcentrifugation, obtained plasma samples were stored at temperature below −60 °C until analyzed. Ticagrelor and AR-C124910XX plasma concentrations were analyzed with liquid chromatography coupled with tandem mass spectrometry, using a validated method^13,28^. Lower limits of quantification were 4.69 ng/mL for both ticagrelor and AR-C124910XX.

### Statistical analysis

Statistical calculations were performed using the Statistica 12.5 package (StatSoft, Tulsa, OK, USA). Pharmacokinetic calculations and plots were made using the Matlab R2014 software (Mathworks, Natick, MA, USA). Trapezoidal rule was applied to calculate AUC. Data for $\frac{AU{C}_{M}}{AU{C}_{T}}$, age, BMI, creatinine plasma concentration and GFR were presented as means with standard deviations. $\frac{AU{C}_{M}}{AU{C}_{T}}$ was evaluated for the period from 0 to 6 hours. Continuous variables were compared between both study arms with the Student’s t-test and Mann–Whitney U test, depending on the presence or absence of the normal distribution. To determine variables associated with changed $\frac{AU{C}_{M}}{AU{C}_{T}}$ values, we performed a linear regression analysis for each feature listed in Table 1. A subsequent multiple regression analysis included characteristics that were found to significantly alter $\frac{AU{C}_{M}}{AU{C}_{T}}$. Finally, a supplementary multiple regression analysis was performed to assess the impact on $\frac{AU{C}_{M}}{AU{C}_{T}}$ exerted by clinical variables used for the main multiple regression analysis in addition with variables that were suggested by the literature to affect metabolism of ticagrelor. In all cases, two-sided p-values < 0.05 were considered significant.

### Data availability

The dataset from NCT02602444 trial analyzed during the current study is available in the figshare.com repository (doi:10.6084/m9.figshare.5396989). The dataset from NCT02612116 trial analyzed during the current study are available from the corresponding author on reasonable request.

## Results

Mean $\frac{AU{C}_{M}}{AU{C}_{T}}\,\,$for the whole analyzed population was 20.4 ± 10.4%. A linear regression analysis of clinical variables listed in Table 1 revealed that diagnosis of STEMI, presence of DM and co-administration of morphine are independent predictors of lower $\frac{AU{C}_{M}}{AU{C}_{T}}$ during the first six hours after ticagrelor LD in ACS patients ($\frac{AU{C}_{M}}{AU{C}_{T}}$ in STEMI: −3.7%, 95% confidence interval [CI] −5.5% to −1.8%, p = 0.0001; $\frac{AU{C}_{M}}{AU{C}_{T}}$ in DM: −2.3%, 95% CI −4.6% to −0.03%, p = 0.048; $\frac{AU{C}_{M}}{AU{C}_{T}}$ in patients co-administered with morphine: −3.0%, 95% CI −5.0% to −1.0%, p = 0.004) (Table 2).

**Table 2:** The impact of clinical features on the rate of ticagrelor metabolism according to linear regression analyses for each variable.

	Regression coefficient	95% confidence interval	p value	R^2^
Age (per year)	0.02	−0.17; 0.21	0.84	0.02
Female	0.04	−1.97; 2.05	0.97	<0.001
BMI (per kg/m^2^)	−0.37	−0.81; 0.06	0.09	0.16
Obesity (BMI ≥ 30 kg/m^2^)	−1.33	−3.49; 0.83	0.22	0.01
Creatinine at admission (per mg/dL)	−3.83	−14.08; 6.42	0.46	0.07
GFR at admission (per mL/minute)	0.05	−0.07; 0.17	0.42	0.07
STEMI	−3.67	−5.51; −1.83	0.0001	0.12
Integral ticagrelor tablets (vs. crushed ticagrelor tablets)	−2.05	−4.19; 0.09	0.06	0.03
Morphine administration	−3.04	−5.04; −0.98	0.004	0.07
History of CAD	0.39	−1.70; 2.49	0.71	0.001
Nonhemorrhagic stroke	0.10	−3.50; 3.69	0.96	<0.001
LVEF at discharge < 50%	−1.22	−3.12; 0.68	0.21	0.01
Hypertension	0.55	−1.43; 2.54	0.58	<0.01
Diabetes mellitus	−2.32	−4.62; −0.03	0.048	0.03
Dyslipidemia	−0.17	−3.22; 2.88	0.91	<0.001
Current smoker	0.63	−1.36; 2.62	0.53	<0.01

BMI: body mass index, CAD: coronary artery disease, GFR: glomerular filtration rate, LVEF: left ventricle ejection fraction, STEMI: ST-elevation myocardial infarction.

Furthermore, a multiple regression analysis confirmed that both diagnosis of STEMI (−6.8%, 95% CI −10.9% to −2.8%, p < 0.001) and DM (−5.5%, 95% CI −9.8% to −1.2%, p = 0.013), but not use of morphine (p = 0.22), are connected with lower $\frac{AU{C}_{M}}{AU{C}_{T}}$ (Table 3). An additional, exploratory multiple regression analysis of variables included in the main multiple regression analysis together with age, gender, GFR and smoking status has been performed due to available data from another studies suggesting that these characteristics may also affect the degree of ticagrelor metabolism into AR-C124910XX^29–31^. This supplementary analysis again showed that presence of STEMI (−8.2%, 95% CI −12.4% to −4.0%, p = 0.0002) and DM (−5.9%, 95% CI −10.3% to −1.6%, p = 0.008) were related to lower $\frac{AU{C}_{M}}{AU{C}_{T}}$. Moreover, it also revealed that smoking patients had greater degree of transformation of ticagrelor into its active metabolite (4.2%, 95% CI 0.2% to 8.3%, p = 0.04), while lack of relationship was found for age, gender, morphine administration and GFR (Table 4). The R^2^ value of 0.18 for the primary and of 0.22 for supplementary multiple regression analysis indicated that 18% and 22% of the variability in $\frac{AU{C}_{M}}{AU{C}_{T}}$ can be explained by each model, respectively.

**Table 3:** The impact of clinical features on the rate of ticagrelor metabolism according to the main multiple regression analysis.

	Regression coefficient	Standard error	95% confidence interval	p value	R^2^ (overall significance)
Constant	25.0	1.3	22.5; 27.6	0.0001	0.18 (0.000045)
STEMI	−6.8	2.0	−10.9; −2.8	0.0009	
Morphine co-administration	−2.7	2.8	−7.0; 1.7	0.22	
Diabetes mellitus	−5.5	2.2	−9.8; −1.2	0.013	

STEMI: ST-elevation myocardial infarction.

**Table 4:** The impact of clinical features on the rate of ticagrelor metabolism according to the additional multiple regression analysis.

Parameter	Regression coefficient	Standard error	95% confidence interval	p value	R^2^ (overall significance)
Constant	17.1	7.2	2.8; 31.4	0.02	0.21 (0.00014)
Age	0.1	0.1	−0.1; 0.3	0.36	
Gender	1.6	2.0	−2.3; 5.4	0.43	
STEMI	−8.2	2.1	−12.4; −4.0	0.0002	
Morphine co-administration	−2.2	2.2	−6.6; 2.1	0.31	
Current smoker	4.2	2.0	0.2; 8.3	0.04	
Diabetes mellitus	−5.9	2.2	−10.3; −1.6	0.008	

STEMI: ST-elevation myocardial infarction.

**Figure 3:**
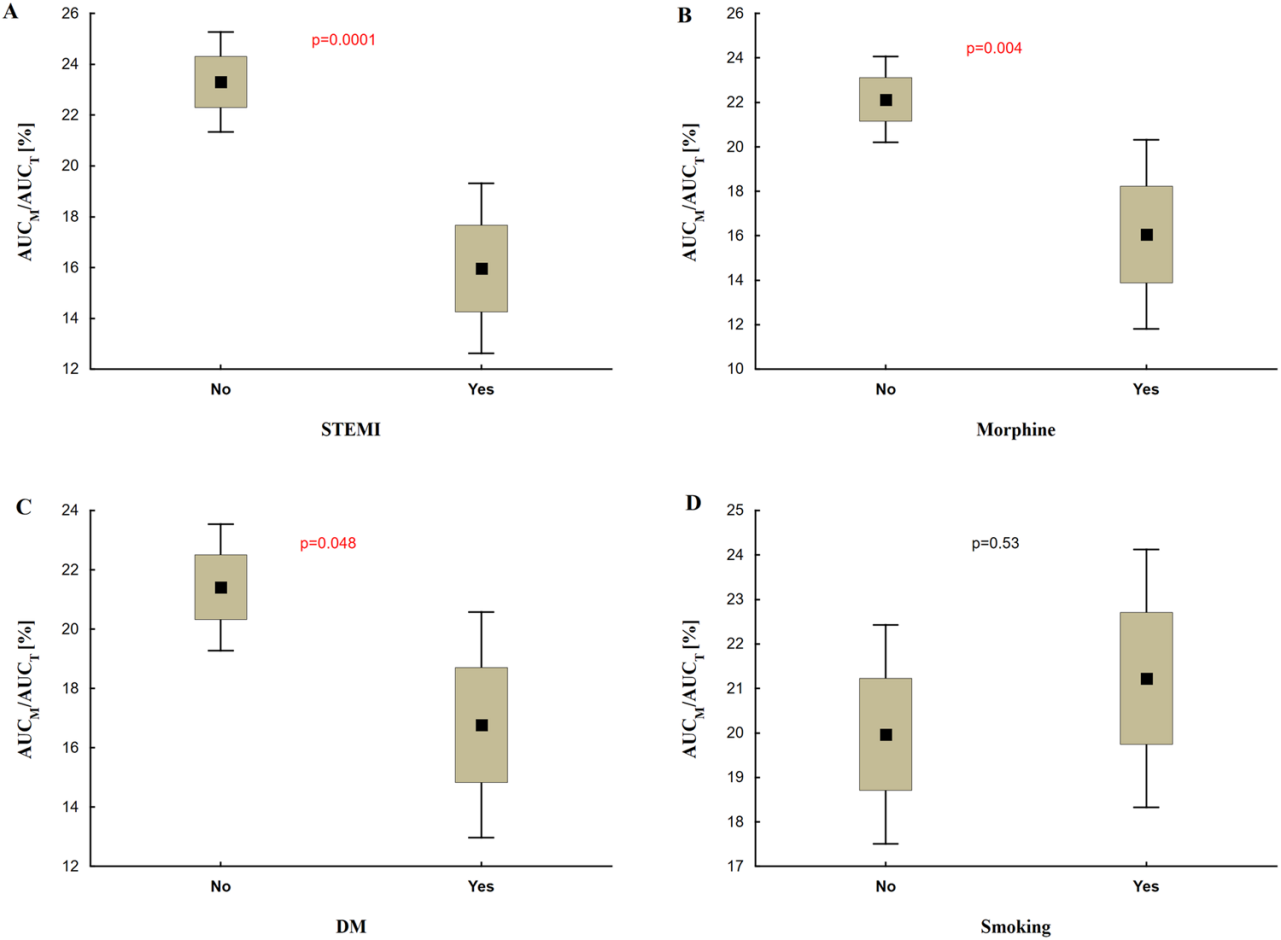
AR-C124910XX to ticagrelor plasma concentration ratios. Comparison of metabolite to parent drug concentration ratios based on the presence of STEMI (vs. NSTE-ACS) (**A**), morphine administration (**B**), DM (**C**) or smoking status (**D**). AUC_M_/AUC_T_: the ratio of area under the plasma concentration-time curve for AR-C124910XX to area under the plasma concentration-time curve for ticagrelor; DM: diabetes mellitus; NSTE-ACS: non-ST-elevation acute coronary syndrome; STEMI: ST-elevation myocardial infarction.

In a direct comparison we found that patients with STEMI, DM or those treated with morphine had significantly lower $\frac{AU{C}_{M}}{AU{C}_{T}}$ compared with patients with NSTE-ACS, non-diabetic or morphine-naive patients, respectively (STEMI vs. NSTE-ACS: 16.0% ± 11.6% vs. 23.3% ± 8.5%, p = 0.0001; morphine vs. morphine-naive: 16.1% ± 12.5% vs. 22.1% ± 9.0%, p = 0.004; DM vs. non-DM: 16.8% ± 9.7% vs. 21.4% ± 10.4%, p = 0.048). When we compared $\frac{AU{C}_{M}}{AU{C}_{T}}$ for smokers and non-smokers the difference between the groups was not significant (21.2% ± 9.6% vs. 20.0% ± 10.9%; p = 0.53) (Fig. 3).

## Discussion

Our analysis of patient-level pharmacokinetic data from two prospective trials revealed that ACS patients presenting with STEMI or suffering from DM are more likely to have decreased ticagrelor metabolism rate during the acute phase of ACS. Analgesic treatment with morphine in this setting was found to negatively influence transformation of ticagrelor into AR-C124910XX when assessed with linear regression analysis, but this effect was not significant anymore when evaluated with multiple regression analyses. On the other hand, smoking appears to increase the degree of ticagrelor transformation expressed as $\frac{AU{C}_{M}}{AU{C}_{T}}$ in ACS patients. Our study shows that increased bioavailability of crushed ticagrelor tablets does not influence the rate of AR-C124910XX formation following ticagrelor LD in ACS patients^25,32,33^. Admittedly, all STEMI patients included in our study received integral ticagrelor tablets, therefore results of our linear regression analysis in regard to ticagrelor pill formulation may not be applicable to this ACS subtype and require further investigation.

Excessive activation of thrombocytes is one of the main underlying pathomechanisms of ACS. High platelet reactivity (HPR) is a risk factor for adverse thrombotic events in ACS patients, including stent thrombosis, a potentially fatal complication of PCI^2^. Moreover, HPR can also be associated with increased mortality in ACS patients^34^. Thus, obtaining a quick and powerful platelet blockade is necessary from the very early stage of ACS treatment, even in conservatively managed patients. Ticagrelor, the first line P2Y12 receptor inhibitor in ACS, thanks to its favorable pharmacokinetics provides a strong antiplatelet action swiftly after oral ingestion. It has to be noted though, that ticagrelor absorption and onset of action are delayed in patients with ACS compared with healthy volunteers or patients with stable coronary artery disease.

Although ticagrelor is responsible for the majority of the observed antiplatelet effect, its active metabolite is equipotent in P2Y12 receptor inhibition and also plays an important role in limiting excessive platelet activation^9^. Ticagrelor is metabolized and transformed into AR-C124910XX predominantly by CYP3A4 and CYP3A5 enzymes^18^. Therefore, metabolism of ticagrelor is affected by concomitant administration of strong CYP3A inducers or inhibitors. CYP3A inducers may essentially decrease total exposure to ticagrelor and its active metabolite even by 86% and 46%, respectively, as seen in subjects receiving rifampicin^19^. On the other hand, strong CYP3A4 inhibitors, for example ketoconazole, may increase ticagrelor exposure by 632%, simultaneously decreasing bioavailability of AR-C124910XX by 56%^20^. Due to the described drug-drug interactions, use of ticagrelor together with powerful CYP3A4 inducers and inhibitors is contraindicated. On the other hand, although certain genetic variants have been shown to modestly affect ticagrelor and AR-C124910XX pharmacokinetics in the setting of ACS, they were not associated with any detectable effect on efficacy or safety during ticagrelor treatment^35^.

The impact of different clinical characteristics on ticagrelor pharmacokinetics has been evaluated in several previous trials. A study by Teng *et al*. showed that in healthy volunteers the overall exposure to AR-C124910XX after a single 200 mg loading dose of ticagrelor was 37% higher in women than in men, and 48% higher in elderly than in young individuals, however these differences were proportional to ticagrelor bioavailability between the groups^29^. In a different trial, patients with severe renal impairment (GFR < 30 mL/min) compared with volunteers with normal renal function (GFR > 80 mL/min) had 20% lower ticagrelor and 17% higher AR-C124910XX bioavailability following a single 180 mg LD of ticagrelor. Subsequently, patients with impaired renal function had 48% higher metabolite to parent ratio for AUC^30^. Butler and Teng also demonstrated that patients with severe hepatic impairment (Child-Pugh class A) had 23% and 66% higher total bioavailability of ticagrelor and its metabolite, respectively, than volunteers with normal liver function after single 90 mg ticagrelor dose^36^. Moreover, population pharmacokinetic model developed for ticagrelor indicated that smoking decreases apparent ticagrelor clearance, simultaneously increasing apparent clearance of AR-C124910XX^31^. Despite the pharmacokinetic differences observed in the aforementioned studies a substantial platelet inhibition was obtained regardless of gender, age, presence of severe renal or mild hepatic impairment, or smoking status, and no adjustment in ticagrelor dose was considered necessary in the described populations^11,29,30,36^. Nevertheless, it has to be underlined that these observations were made based on the results obtained from individuals without ACS. Recent pharmacokinetic and pharmacodynamic studies clearly show that presence of ACS, and especially STEMI, delays and decreases bioavailability, onset of action and antiplatelet effect of ticagrelor^13–16,37^. It is established that presence of HPR, which may be caused by impaired ticagrelor pharmacokinetics, is related to adverse thrombotic events in patients with ACS^2,34^.

In subanalysis of the IMPRESSION study we have reported that although morphine administration did not affect $\frac{AU{C}_{M}}{AU{C}_{T}}$ in patients with AMI, the observed $\frac{AU{C}_{M}}{AU{C}_{T}}$ values were lower than these reported for healthy volunteers (21–25% vs. 35–40%)^8,9,21^. Similarly, in the current analysis the mean $\frac{AU{C}_{M}}{AU{C}_{T}}$ was approximately 20% for the whole study population. It could be speculated that it is due to delayed absorption of ticagrelor in ACS combined with relatively short window of observation (the first six hours after ticagrelor LD) compared with trials in healthy volunteers.

In line with previous studies, the results of the current analysis show that presence of STEMI essentially disturbs pharmacokinetic profile of ticagrelor^13,14,16^. From all examined clinical features STEMI had the greatest negative impact on $\frac{AU{C}_{M}}{AU{C}_{T}}$ (beta-coefficient for supplementary multiple regression analysis = −0.38). Our results show that the mechanism of impaired ticagrelor pharmacokinetics seen in patients with STEMI during the initial hours after the LD may be multifactorial and related not only to slower intestinal absorption, but also to diminished hepatic metabolism. Interestingly, presence of DM was also found to have significant negative influence on $\frac{AU{C}_{M}}{AU{C}_{T}}$ in all performed analyses (beta-coefficient for supplementary multiple regression analysis = −0.24). DM is one of the most important and most common risk factors for ACS. It has to be considered that the effect of DM on $\frac{AU{C}_{M}}{AU{C}_{T}}$ can further aggravate the effect of STEMI, and that the co-existence of these two conditions is not rare. Admittedly, the mechanisms behind the effect of STEMI or DM on $\frac{AU{C}_{M}}{AU{C}_{T}}$ remain unknown.

The impact of the remaining evaluated features on ticagrelor hepatic transformation during the first six hours after ticagrelor LD is ambiguous. Morphine, which delays and attenuates bioavailability of ticagrelor in patients with AMI^13^, in our analysis significantly decreased $\frac{AU{C}_{M}}{AU{C}_{T}}$ only when assessed with linear regression analysis. Morphine follows metabolic pathway which does not include CYP3A enzymes, thus clinically relevant interaction with ticagrelor metabolism is rather unlikely^38^. Our supplementary multiple regression analysis model included smoking as a potential factor influencing ticagrelor metabolism, based on a pharmacokinetic model for ticagrelor and its metabolite suggesting that smoking alters apparent clearance of ticagrelor and AR-C124910XX^31^. In our analysis habitual smoking was the only clinical characteristic that positively affects $\frac{AU{C}_{M}}{AU{C}_{T}}$ (beta-coefficient for supplementary multiple regression analysis = 0.20). The mechanism behind this finding also remains undetermined as smoking does not affect CYP3A-mediated metabolism^31^.

In subgroup analyses of the PLATO study the cardiovascular benefit was consistent in patients treated with ticagrelor compared with those treated with clopidogrel irrespective of type of treatment for STEMI or NSTE-ACS, diabetic status and glycemic control, or smoking status^39–42^. Thus, the clinical significance of our findings is obscure, uncertain and warrants further research.

### Study limitations

One could perceive lack of pharmacodynamic data as a limitation of this analysis, however it was the authors’ intention to focus on ticagrelor metabolism only. It also has to be noted that the ethnicity of the examined group was homogenous (100% Caucasian), which closely reflects the ethnical structure seen in Poland, where both analyzed studies were conducted. Additionally, smoking status was collected only based on information obtained from each patient, and was not validated objectively.

Moreover, several limitations resulted from the design of the included trials. Neither of two studies evaluated clinical endpoints, thus these data were not available. The information on the time span between the onset of chest pain and ticagrelor loading dose was incomplete, and therefore was not included in the analysis. The liver function was not evaluated at the time of enrolment for the analyzed studies, and the exclusion due to liver function impairment was made based on medical history of severe hepatic disease only.

## Conclusions

The degree of ticagrelor hepatic transformation into its active metabolite following a standard 180 mg LD in the setting of ACS is decreased in patients with STEMI and DM. Morphine may be related to smaller, and smoking with greater rate of ticagrelor metabolism in patients with ACS, but these data are not completely conclusive.

## References

[1] Lange RA, Hillis LD (2004). Antiplatelet therapy for ischemic heart disease. N Engl J Med..

[2] Brar SS (2011). Impact of platelet reactivity on clinical outcomes after percutaneous coronary intervention. A collaborative meta-analysis of individual participant data. J Am Coll Cardiol..

[3] Ibanez B (2018). 2017 ESC Guidelines for the management of acute myocardial infarction in patients presenting with ST-segment elevation: The Task Force for the management of acute myocardial infarction in patients presenting with ST-segment elevation of the European Society of Cardiology (ESC). Eur Heart J..

[4] Roffi M (2016). 2015 ESC Guidelines for the management of acute coronary syndromes in patients presenting without persistent ST-segment elevation: Task Force for the Management of Acute Coronary Syndromes in Patients Presenting without Persistent ST-Segment Elevation of the European Society of Cardiology (ESC). Eur Heart J..

[5] O’Gara PT (2013). 2013 ACCF/AHA guideline for the management of ST-elevation myocardial infarction: a report of the American College of Cardiology Foundation/American Heart Association Task Force on Practice Guidelines. Circulation..

[6] Amsterdam EA (2014). 2014 AHA/ACC guideline for the management of patients with non-ST-elevation acute coronary syndromes: a report of the American College of Cardiology/American Heart Association Task Force on Practice Guidelines. Circulation..

[7] Adamski P (2014). Overview of pleiotropic effects of platelet P2Y12 receptor inhibitors. Thromb Haemost..

[8] Husted S (2006). Pharmacodynamics, pharmacokinetics, and safety of the oral reversible P2Y12 antagonist AZD6140 with aspirin in patients with atherosclerosis: a double-blind comparison to clopidogrel with aspirin. Eur Heart J..

[9] Teng R, Oliver S, Hayes MA, Butler K (2010). Absorption, distribution, metabolism, and excretion of ticagrelor in healthy subjects. Drug Metab Dispos..

[10] Teng R, Butler K (2010). Pharmacokinetics, pharmacodynamics, tolerability and safety of single ascending doses of ticagrelor, a reversibly binding oral P2Y(12) receptor antagonist, in healthy subjects. Eur J Clin Pharmacol..

[11] Husted SE (2012). Pharmacokinetics and pharmacodynamics of ticagrelor in patients with stable coronary artery disease: results from the ONSET-OFFSET and RESPOND studies. Clin Pharmacokinet..

[12] Teng R (2015). Ticagrelor: Pharmacokinetic, Pharmacodynamic and Pharmacogenetic Profile: An Update. Clin Pharmacokinet..

[13] Kubica J (2016). Morphine delays and attenuates ticagrelor exposure and action in patients with myocardial infarction: the randomized, double-blind, placebo-controlled IMPRESSION trial. Eur Heart J..

[14] Adamski P (2017). Comparison of bioavailability and antiplatelet action of ticagrelor in patients with ST-elevation myocardial infarction and non-ST-elevation myocardial infarction: a prospective, observational, single-centre study. PLoS One..

[15] Koziński M (2016). Which platelet function test best reflects the *in vivo* plasma concentrations of ticagrelor and its active metabolite? The HARMONIC study. Thromb Haemost..

[16] Franchi F (2015). Impact of Escalating Loading Dose Regimens of Ticagrelor in Patients With ST-Segment Elevation Myocardial Infarction Undergoing Primary Percutaneous Coronary Intervention: Results of a Prospective Randomized Pharmacokinetic and Pharmacodynamic Investigation. JACC Cardiovasc Interv..

[17] Kubica J (2016). Impact of morphine on antiplatelet effects of oral P2Y12 receptor inhibitors. Int J Cardiol..

[18] Zhou D, Andersson TB, Grimm SW (2011). *In vitro* evaluation of potential drug-drug interactions with ticagrelor: cytochrome P450 reaction phenotyping, inhibition, induction, and differential kinetics. Drug Metab Dispos..

[19] Teng R, Mitchell P, Butler K (2013). Effect of rifampicin on the pharmacokinetics and pharmacodynamics of ticagrelor in healthy subjects. Eur J Clin Pharmacol..

[20] Teng R, Butler K (2013). Effect of the CYP3A inhibitors, diltiazem and ketoconazole, on ticagrelor pharmacokinetics in healthy volunteers. J Drug Assess..

[21] Adamski P (2015). Does morphine administration affect ticagrelor conversion to its active metabolite in patients with acute myocardial infarction? A sub-analysis of the randomized, double-blind, placebo-controlled IMPRESSION trial. Folia Med Copernicana..

[22] Siller-Matula JM, Trenk D, Krähenbühl S, Michelson AD, Delle-Karth G (2014). Clinical implications of drug-drug interactions with P2Y12 receptor inhibitors. J Thromb Haemost..

[23] Adamski P (2017). Comparison of Ticagrelor Pharmacokinetics and Pharmacodynamics in STEMI and NSTEMI Patients (PINPOINT): protocol for a prospective, observational, single-centre study. BMJ Open..

[24] Niezgoda P (2016). Impact of ticagrelor administration strategy on its pharmacokinetics and pharmacodynamics in patients with unstable angina pectoris: a protocol of a randomized study. Med Res J..

[25] Niezgoda P (2017). Crushed sublingual versus oral ticagrelor administration strategies in patients with unstable angina. A pharmacokinetic/pharmacodynamic study. Thromb Haemost..

[26] Thygesen K (2012). Third universal definition of myocardial infarction. Eur Heart J..

[27] Steg PG (2012). ESC Guidelines for the management of acute myocardial infarction in patients presenting with ST-segment elevation. Eur Heart J..

[28] Sillén H, Cook M, Davis P (2010). Determination of ticagrelor and two metabolites in plasma samples by liquid chromatography and mass spectrometry. J Chromatogr B Analyt Technol Biomed Life Sci..

[29] Teng R, Mitchell P, Butler K (2012). Effect of age and gender on pharmacokinetics and pharmacodynamics of a single ticagrelor dose in healthy individuals. Eur J Clin Pharmacol..

[30] Butler K, Teng R (2012). Pharmacokinetics, pharmacodynamics, and safety of ticagrelor in volunteers with severe renal impairment. J Clin Pharmacol..

[31] Li J, Tang W, Storey RF, Husted S, Teng R (2016). Population pharmacokinetics of ticagrelor in patients with acute coronary syndromes. Int J Clin Pharmacol Ther..

[32] Parodi G (2015). Ticagrelor crushed tablets administration in STEMI patients: the MOJITO study. J Am Coll Cardiol..

[33] Alexopoulos D (2016). Crushed Versus Integral Tablets of Ticagrelor in ST-Segment Elevation Myocardial Infarction Patients: A Randomized Pharmacokinetic/Pharmacodynamic Study. Clin Pharmacokinet..

[34] Aradi D (2015). Bleeding and stent thrombosis on P2Y12-inhibitors: collaborative analysis on the role of platelet reactivity for risk stratification after percutaneous coronary intervention. Eur Heart J..

[35] Varenhorst C (2015). Effect of genetic variations on ticagrelor plasma levels and clinical outcomes. Eur Heart J..

[36] Butler K, Teng R (2011). Pharmacokinetics, pharmacodynamics, and safety of ticagrelor in volunteers with mild hepatic impairment. J Clin Pharmacol..

[37] Parodi G (2013). Comparison of prasugrel and ticagrelor loading doses in ST-segment elevation myocardial infarction patients: RAPID (Rapid Activity of Platelet Inhibitor Drugs) primary PCI study. J Am Coll Cardiol..

[38] Smith HS (2009). Opioid metabolism. Mayo Clin Proc..

[39] Steg PG (2010). Ticagrelor versus clopidogrel in patients with ST-elevation acute coronary syndromes intended for reperfusion with primary percutaneous coronary intervention: A Platelet Inhibition and Patient Outcomes (PLATO) trial subgroup analysis. Circulation..

[40] Lindholm D (2014). Ticagrelor vs. clopidogrel in patients with non-ST-elevation acute coronary syndrome with or without revascularization: results from the PLATO trial. Eur Heart J..

[41] James S (2010). Ticagrelor vs. clopidogrel in patients with acute coronary syndromes and diabetes: a substudy from the PLATelet inhibition and patient Outcomes (PLATO) trial. Eur Heart J..

[42] Cornel JH (2012). Prior smoking status, clinical outcomes, and the comparison of ticagrelor with clopidogrel in acute coronary syndromes-insights from the PLATelet inhibition and patient Outcomes (PLATO) trial. Am Heart J..

